# Applying perceptual learning to achieve practical changes in vision

**DOI:** 10.3389/fpsyg.2014.01166

**Published:** 2014-10-16

**Authors:** Jenni Deveau, Aaron R. Seitz

**Affiliations:** Department of Psychology, University of California RiversideRiverside, CA, USA

**Keywords:** perceptual learning, presbyopia, visual therapy, applied vision, reading

## Abstract

Research of visual perceptual learning has illuminated the flexibility of processing in the visual system and provides insights into therapeutic approaches to remediating some components of low vision. A key observation from research of perceptual learning is that effects of training are often highly specific to the attributes of the trained stimuli. This observation has been a blessing to basic research, providing important constraints to models of learning, but is a curse to translational research, which has the goal of creating therapies that generalize widely across visual tasks and stimuli. Here we suggest that the curse of specificity can be overcome by adopting a different experimental framework than is standard in the field. Namely, translational studies should integrate many approaches together and sacrifice mechanistic understanding to gain clinical relevance. To validate this argument, we review research from our lab and others, and also present new data, that together shows how perceptual learning on basic stimuli can lead to improvements on standard vision tests as well as real world vision use such as improved reading and even improved sports performance. Furthermore, we show evidence that this integrative approach to perceptual learning can ameliorate effects of presbyopia and provides promise to improve visual function for individuals suffering from low vision.

## INTRODUCTION

Vision is a highly beneficial sense that is paramount to social interactions, navigation of the world, and most workplace and leisure activities. As such, poor vision can have profound negative impact on peoples’ ability to interact with the world around them. Acknowledging this problem there is a tremendous industry associated with optical devises, surgical procedures, specialized drugs, etc with a focus on improving the operation of the eye. However, our ability to see relies not only on a well-functioning eye with good optics, but also on how the brain makes use of this information. Many examples of poor vision, such as due to strokes, traumatic brain damage, or developmental disorders such as amblyopia, make clear that impaired brain processing is an important component of low vision. Furthermore, as we argue in the present paper, suboptimal brain processing of visual information is the norm and that there is a need to develop therapies that address the brain’s contribution to poor vision. These “brain training” approaches have the potential to ameliorate impacts of retinal disease, potentially cure conditions of cortical dysfunction such as amblyopia, and unlock substantial gains for normally functioning individuals, across the life-span.

Key examples of the ability of the adult visual system to improve processing come from the field of perceptual learning (Sagi, 2011). Perceptual learning is often defined as improvements in performance on visual tasks following practice or experience with stimuli related to those tasks and has been a focus of substantial research over the last 40 years. By now, practically any visual skill that can be described has been the target of at least some study of perceptual learning (Fahle and Poggio, 2002) and collectively research of perceptual learning demonstrates that there is room for improvement in most aspects of vision. Furthermore, perceptual learning research is exemplified by the long-lasting improvement on simple but difficult perceptual tasks with benefits shown to last months, even years (Ball and Sekuler, 1981; Sagi and Tanne, 1994; Crist et al., 2001).

Given the demonstrated plasticity of the visual system and the longevity of benefits, one would assume wide-scale adoption of perceptual learning approaches in clinical settings. However, despite the plethora of research, perceptual learning research has had limited penetration into the clinic. While there are many reasons for this, such as most research of perceptual learning is from Psychology and Neuroscience, having limited interactions with Optometrists and Ophthalmologists, and with most perceptual learning research mostly involving normally seeing human subjects or animals, with limited research in low vision populations. In addition, research of perceptual learning has been dominated by, and in some case defined by, examples of learning that are specific to the particulars of the stimuli experienced during training; trained stimulus features (Fahle, 2005), such as orientation (Fiorentini and Berardi, 1980), motion direction (Ball and Sekuler, 1981; Watanabe et al., 2002), retinal location (Karni and Sagi, 1991) or even the trained eye (Poggio et al., 1992; Seitz et al., 2009). While such findings provide insights into the brain system that underlie perceptual learning, and help constrain models of perceptual learning, training that only manifests at a single retinal location, for a limited stimulus space, provides limited therapeutic benefit. As such specificity, which is a “blessing” to mechanistic studies of perceptual learning, is a “curse” to clinical viability.

However, there is increasing evidence that certain types of training yields beneficial learning that transfer beyond the trained context. Notable examples include vision training to improve reading (Chung et al., 2004), or hitting baseballs (Deveau et al., 2014b). Furthermore, numerous studies suggest that perceptual learning can lead to relatively broad-based improvements in visually impaired individual such as amblyopia (Levi and Li, 2009), peripheral vision loss (Chung, 2011), presbyopia (Polat, 2009), macular degeneration (Baker et al., 2005), stroke (Huxlin et al., 2009; Das et al., 2014), and late-life recovery of visual function (Ostrovsky et al., 2006) and other individuals with impaired vision (Huang et al., 2008; Zhou et al., 2012). These studies suggest the potential value of perceptual learning as a rehabilitative approach for individuals with low vision and that the curse of specificity can be overcome.

## OVERCOMING THE CURSE OF SPECIFICITY

Specificity perceptual learning stems, at least in part, from research procedures that train participants on reduced stimulus sets (e.g., single orientation at single retinotopic location). Such training engages a limited neural population (Fahle, 2004), teaches participants to attend to this limited features space and to ignore other features (Zhang et al., 2013), and encourages decision policies/strategies that will be specific to this limited feature space (Fulvio et al., 2014). While there exists substantial debate regarding which neural mechanisms underlie specificity (Dosher and Lu, 1998; Fahle, 2004; Hung and Seitz, 2014), it is arguable that specificity occurs due to some form of overfitting of the training task (Mollon and Danilova, 1996; Sagi, 2011).

Training regimes that employ a broader stimulus space, such as those using multi-stimulus training (Xiao et al., 2008; Yu et al., 2010; Deveau et al., 2014a,b) and off-the-shelf video games (Green and Bavelier, 2003; Li et al., 2009) show greater generalization of learning than typically found in studies of perceptual learning. For example, Xiao et al. (2008) trained participants on a Vernier discrimination task at a specific orientation and retinotopic location, which classically leads retinotopic and orientation specific learning (Poggio et al., 1992), however, after training on a second orientation at a different spatial location, learning transferred across locations (although see Hung and Seitz, 2014). Taking this approach to clinical populations, Das et al. (2014) used a double training procedure where static and dynamic stimuli were presented to patients with cortical blindness in separate retinotopic locations. They found training with complex moving stimuli at one location transferred to improvements in a location only trained with static stimuli. Growing research shows how a diversity of factors can contribute to overcoming the curse of specificity; for example, the amount of training (Aberg et al., 2009; Jeter et al., 2010), and the difficulty/precision of the stimulus judgments training (Ahissar and Hochstein, 1997; Hung and Seitz, 2014) or testing (Jeter et al., 2009).

## INTEGRATING MULTIPLE APPROACHES TO ACHIEVE GREATER LEARNING

We hypothesized that the greatest degree of learning and broadest transfer could be achieved by combining approaches from different research studies targeting different perceptual learning mechanisms. To test this hypothesis we combined multiple perceptual learning approaches, including training with a diverse set of stimuli (Xiao et al., 2008), optimized stimulus presentation (Beste et al., 2011), multisensory facilitation (Shams and Seitz, 2008), and consistent reinforcement of training stimuli (Seitz and Watanabe, 2009), which have individually contributed to increasing the speed (Seitz et al., 2006), magnitude (Seitz et al., 2006; Vlahou et al., 2012), and generality of learning (Green and Bavelier, 2007; Xiao et al., 2008) into a simple video game (for details see Deveau et al., 2014a,b) that trained a diverse set of stimuli (multiple orientations, spatial frequencies, locations, distractor types, etc).

**FIGURE 1:**
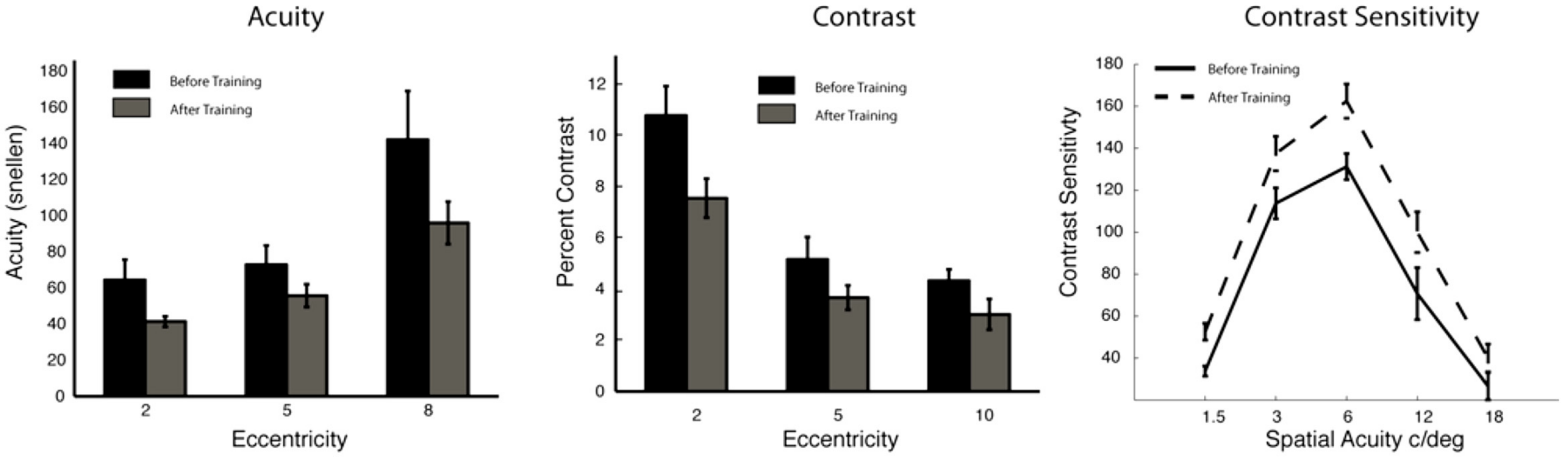
**Data from Deveau et al. (2014a). Left**, for acuity, Landolt C size thresholds were measured at different locations in the visual field (with an eye-tracker to enforce fixation). **Middle**, contrast sensitivity thresholds were measured by varying the contrast of an “O” presented at visual field locations. **Right**, an Optec Visual Analyzer (Stereo Optical Company, Chicago, IL, USA) measured foveal visual acuity and contrast sensitivity. Data from pre-training tests (black) is shown against data of post-training tests (gray). In the left two graphs, lower values represent better performance. Acuity values **(left)** are based on standard 20/20 scores in the fovea (peripheral scores values are poorer). Weber Contrast **(middle)**. Contrast Sensitivity **(right)** shows contrast as a function of spatial frequency in central vision (higher values are better). Training-induced benefits are all significant at least to the *p*< 0.05 levels. Error bars represent SE of the mean.

Initial research using this integrated perceptual learning game provides support for our hypothesis of the effectiveness of this approach. In a first study (Deveau et al., 2014a), 14 participants (age 18–55) completed 24 training sessions and conducted tests of visual acuity and contrast sensitivity before and after training. Results showed significant improvements to central and peripheral vision (see **Figure 1**). Following up on this work, we investigated the extent to which such visual training could impact performance in the daily activities of study participants. To test this, we trained the position players of the UC Riverside baseball team for 30 sessions each with the integrated training game. Results showed both improvements in visual acuity (pre-training Snellen acuity of 20/13 ± 0.69 SE vs. post-training of 20/10 ± 0.59) with seven of the trained players reaching 20/7.5 Snellen acuity after training (Deveau et al., 2014b). Importantly, performance on the baseball field also improved, with trained players showing a significant reduction of strike-outs of 4.4% ± 2.0 SE, and an estimated increase of 41.2 runs created which led to an estimated 4–5 extra games won (over the 54 game season; Deveau et al., 2014b).

## TRANSFER OF INTEGRATED TRAINING TO READING SKILLS

Notably, we also found that near vision was significantly improved in the baseball players after training (Deveau et al., 2014b). This led us to question whether visual abilities related to near vision were improved as well. Given that these were student athletes, we hypothesized that reading skills, which are highly important to the educational goals of the athletes, may also benefit from training.

**FIGURE 2:**
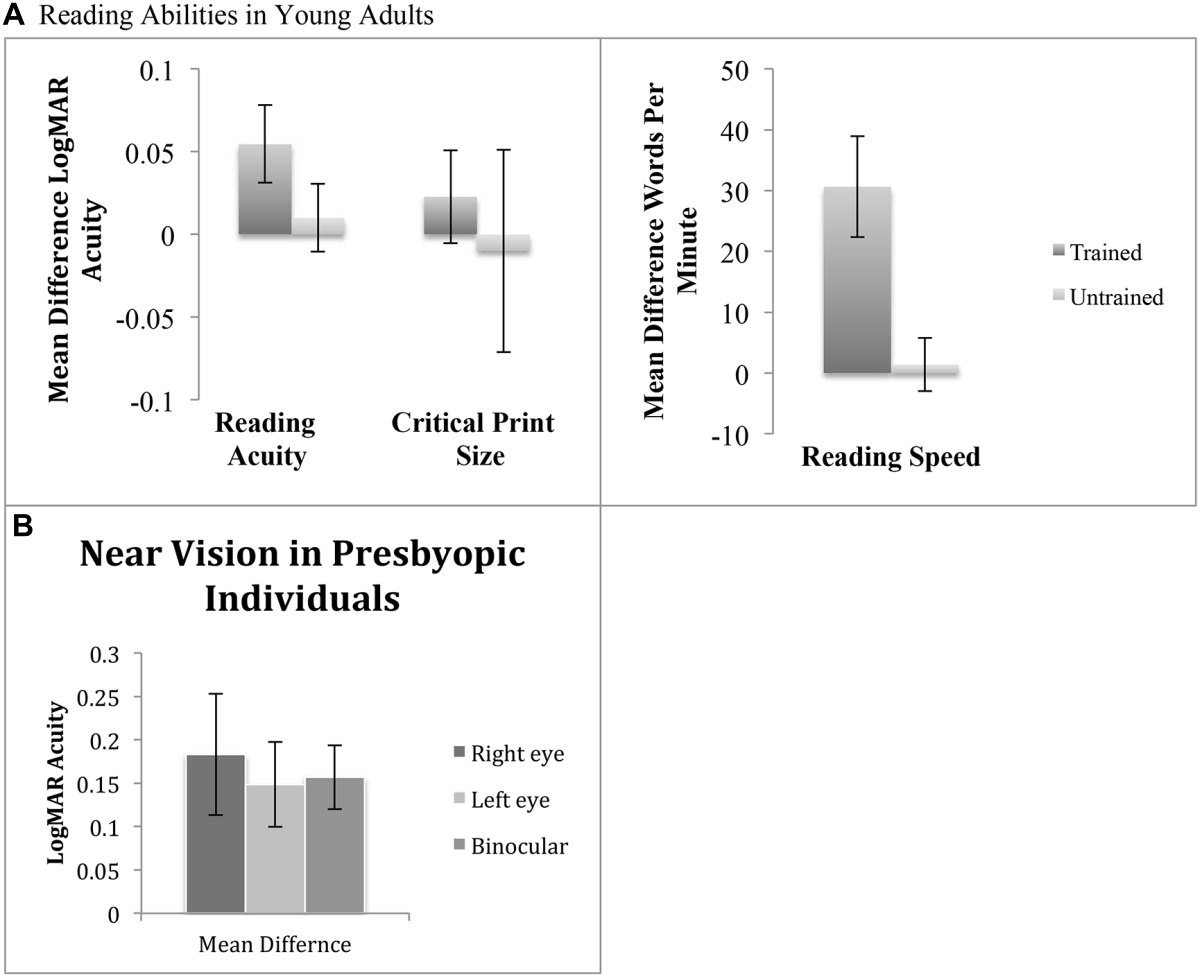
**(A)** Mean difference of reading acuity, critical print size, and reading speed using MNREAD acuity charts in healthy young adults. Trained participants were tested binocularly before and after vision training. Untrained data reproduced from Subramanian and Pardhan (2006) where two different versions of the MNREAD acuity charts were used to measure learning effects. Error bars represent subject SE. **(B)** Mean difference in LogMAR acuity measurements taken in the right eye, left eye, and binocularly in presbyopic individuals. Participants were tested before and after vision training. Error bars represent within subject SE.

To test this hypothesis, we measured reading acuity, speed, and critical print size before and after vision training in 44 UC Riverside undergraduates using MNREAD charts (see **Figure 2A**). The charts contain 19 English sentences (60 characters each) with print sizes ranging from 1.3 to -0.5 logMAR at a distance of 16 inches (0.41 m). Different charts were used for pre and post-tests, all tests were conducted in a well lit room. Charts were placed on a stand 16 inches (0.41 m) away from participants, who were instructed to read each sentence aloud as quickly and as accurately as possible. After each sentence participants would look to the left or right (away from the chart) until instructed to move on to the next sentence by the experimenter. A stopwatch was used to record the time taken to read each sentence to the nearest 0.1 s. The number of errors made in each sentence was also recorded. Reading acuity was calculated as the logMAR of the last sentence read, adding 0.01 logMAR for each reading error. Reading speed was measured in words per minute. Maximum reading speed was calculated as the fastest sentence read, regardless of logMAR. Critical print size was measured as the smallest print size participants can read close to their maximum reading speed.

Vision training consisted of video-game based custom software. The training procedure has been previously described (Deveau et al., 2014a,b). Briefly, the training stimuli consisted of Gabor patches (game “targets”) at six spatial frequencies (1.5, 3, 6.3, 12.5, 25, and 50 cpd), and eight orientations (22.5, 67.5, 112.5, 157.5, 202.5, 247.5, 292.5, or 337.5°). Exercises alternated between static and dynamic types, in the static exercise an array of targets of a single spatial frequency, at a randomly determined orientation were presented randomly on the screen all at once. In the dynamic exercise targets of a randomly determined orientation/spatial frequency combination are presented one at a time, and faded in at a random location on the screen. The goal of the exercises was to click on all the Gabor targets as quickly as possible.

After training, reading acuity improved an average of 13% (*p* = 0.02), moving from a pre training mean logMAR acuity value of -0.06 to a post training value of -0.11, (mean difference SE ± 0.02). Reading speed improved an average of 13% (*p* = 0.0004), moving from a pre training mean value of 240.0 words per minute to a post training value of 270.6 words per minute (mean difference SE ± 8.28). However, critical print size did not improve after training (mean difference = 0.02 logMAR ± 0.03 SE; *t*-test, *p* = 0.40).

While we did not have an untrained control group in this study (see Discussion), previous research has found the MNREAD chart to be resistant to practice effects (**Figure 2A**, untrained). Subramanian and Pardhan (2006) used two different versions of the MNREAD acuity charts to measure learning effects of reading acuity, critical print size, and reading speed. Thirty college age participants conducted assessments in two separate sessions without a training intervention in between. These participants showed no significant differences in reading acuity, critical print size, and reading speed indicating the MNREAD is resistant to practice effects.

These data provide another example of real world benefits to normally seeing young individuals after integrated perceptual learning based vision training. These participants were young, with above average initial acuity. However, as we age near vision declines which can negatively impacting reading. Therefore we next sought to assess practical improvements from vision training in individuals with impaired near vision.

## VISION TRAINING TO AMELIORATE PRESBYOPIA

Presbyopia refers to loss of vision associated with the aging eye and is primarily characterized by a gradual loss of accommodation and reduced elasticity of the lens (von Helmholtz, 1924). The result is the reduced ability to focus at a near distance, a requirement for many daily activities and hobbies such as reading, using a cell phone or tablet, or sewing, which can negatively affects one’s quality of life (McDonnell et al., 2003). This accommodative decline is virtually universal, as of 2005 it is estimated over 1 billion people worldwide are presbyopic, with more than half of individuals without adequate treatment (Holden et al., 2008). The most common form of presbyopia correction is the use of spectacles, including multifocal (bifocal, trifocal, or progressive lens) or reading glasses. There are also several surgical treatment options for presbyopia, however side effects include reduced contrast sensitivity, increased glare, and poor night vision (Papadopoulos and Papadopoulos, 2014) that can negatively impact the overall quality of vision. Given that all other treatments for presbyopia have side-effects, a perceptual learning based approach to ameliorate the impact of presbyopia could have substantial benefits, especially for early stage or mild presbyopia.

Based on this research, here we applied our integrated training program to 13 presbyopic participants (seven male and six females; age range 40–78 years) over the course of 4–12 weeks (average of four sessions per week). Vision training procedures are the same as described in section “Transfer of Integrated Training to Reading Skills.” After training, near vision in participants improved from mean logMAR values of 0.47–0.29 in the right eye (mean difference SE ± 0.07; *t*-test, *p* = 0.01, Pearson *r*= 0.42); 0.45–0.29 in the left eye (mean difference SE ± 0.05; *p* = 0.009, *r* = 0.52); and 0.31–0.15 binocularly (mean difference SE ± 0.04; *p* = 0.0005, *r* = 0.46; see **Figure 2B**). Binocular average initial visual acuity was 0.31 log units and improved to 0.15, a 43% benefit. Four individuals even reached non-presbyopic acuity levels similar to younger individuals, 0.0 logMAR or below.

These data show the promise of perceptual learning based therapies to improve near vision in adults with presbyopia. Our results are consistent with other recent studies showing that perceptual learning can improve contrast sensitivity and near visual acuity in presbyopic individuals (Polat, 2009; Polat et al., 2012). These improvements are unlikely to be the result of simple test–retest improvements, as these were not found in our other control groups and Polat (2009) found improvements in near acuity in presbyopic individuals after training on a contrast detection task, but not in untrained controls. These results provide intriguing potential for the many individuals with presbyopia, however, our current study and previous research (Polat, 2009; Polat et al., 2012) lack the appropriate control conditions to make substantial conclusion. Future studies with a double blind active control group are essential in determining the effectiveness of perceptual learning based vision training.

## DISCUSSION

Classically, perceptual learning leads to learning effects that are specific to the training stimuli, hindering translational progress in alleviating low vision. Here, we suggest that the key to successful translation of perceptual learning research lies upon integrative approaches where the goal is not to achieve highly specific learning, but instead to achieve broad-based improvements to vision. To this end, we combined multiple perceptual learning approaches (including engagement of attention, reinforcement, multisensory stimuli, and multiple stimulus dimensions) that have individually contributed to increasing the speed, magnitude, and generality of learning into an integrated perceptual-learning video game. Training with this video game shows that the “curse of specificity” can be overcome and the perceptual learning based training can lead to improvements in central and peripheral acuity and contrast sensitivity, reading acuity, and speed, and even improved on-field baseball hitting statistics, in normally sighted young adults after training. We also find improvements in near visual acuity in adults with presbyopia; with many of these individuals reaching non-presbyopic acuity levels (0.0 logMAR or below) after training. These data provide evidence perceptual learning based vision training translates to real world skills used in daily life, which is of great practical importance.

While the presented data provide a proof of principle that the integrated vision training program is effective, the lack of a double-blind placebo controlled study raises the possibility of potential placebo effects (Boot et al., 2011). These placebo effects may even be greater in more complex experimental designs, like those used in the current study, where there are multiple factors that might lead participants to believe that their vision should be getting better. Thus while it is classically believed that acuity and contrast sensitivity are relatively robust to placebo effects, and benefits to contrast sensitivity typically require extensive and specialized training (Adini et al., 2002; Furmanski et al., 2004) further works will be required to confirm these results.

Still, the results are consistent with a growing body of research demonstrating improvements in visual abilities after perceptual learning training. For example, other recent successes applying perceptual learning show improvements in subjects with amblyopia (Levi and Li, 2009; Hussain et al., 2012), presbyopia (Polat, 2009), macular degeneration (Baker et al., 2005), stroke (Vaina and Gross, 2004; Huxlin et al., 2009), and late-life recovery of visual function (Ostrovsky et al., 2006) suggest great promise that perceptual learning can ameliorate effects of low vision.

Collectively these studies provide substantial promise for treatment of low vision and improved visual function in normally seeing individuals, alike. However, further research is needed to determine the optimal combination of approaches to improve vision and how these may differ for visual conditions and across individuals. A difficulty towards achieving this end is that most studies in the field, including ours, include small numbers of subjects, limited controls, and substantial individual subject variance. Thus to achieve greater impact in clinical settings the field needs to move towards conducting larger scale exploratory studies to further optimize procedures and clinical trials to further validate effects. While there is thus substantial work required to fully realize the positive impact of perceptual learning based training, we believe that the potential impact to society is substantial.

## Conflict of Interest Statement

Aaron R. Seitz is a founder and stakeholder in Carrot Neurotechnology, which developed the ULTIMEYES program described in the manuscript. This conflict of interest was reviewed and the research approved by the University of California - Riverside Conflict of Interest Committee and the Human Research Review Board.
